# The *SCO1731* methyltransferase modulates actinorhodin production and morphological differentiation of *Streptomyces coelicolor* A3(2)

**DOI:** 10.1038/s41598-018-32027-8

**Published:** 2018-09-12

**Authors:** Annalisa Pisciotta, Angel Manteca, Rosa Alduina

**Affiliations:** 1 Department of Biological, Chemical and Pharmaceutical Sciences and Technologies (STEBICEF), Università degli Studi di Palermo, Viale delle Scienze Bd.16, 90128 Palermo, Italy; 2 Área de Microbiología, Departamento de Biología Funcional and IUOPA, Facultad de Medicina, Universidad de Oviedo, 33006 Oviedo, Spain

## Abstract

*Streptomyces coelicolor* is a Gram-positive microorganism often used as a model of physiological and morphological differentiation in streptomycetes, prolific producers of secondary metabolites with important biological activities. In the present study, we analysed *Streptomyces coelicolor* growth and differentiation in the presence of the hypo-methylating agent 5′-aza-2′-deoxycytidine (5-aza-dC) in order to investigate whether cytosine methylation has a role in differentiation. We found that cytosine demethylation caused a delay in spore germination, aerial mycelium development, sporulation, as well as a massive impairment of actinorhodin production. Thus, we searched for putative DNA methyltransferase genes in the genome and constructed a mutant of the *SCO1731* gene. The analysis of the *SCO1731*::Tn5062 mutant strain demonstrated that inactivation of *SCO1731* leads to a strong decrease of cytosine methylation and almost to the same phenotype obtained after 5-aza-dC treatment. Altogether, our data demonstrate that cytosine methylation influences morphological differentiation and actinorhodin production in *S*. *coelicolor* and expand our knowledge on this model bacterial system.

## Introduction

Base methylation is a DNA modification present in all kingdoms of life, including bacteria. The methylation of cytosines is an important epigenetic mark, well known in higher eukaryotes to control transcriptional regulation that can cause repression or activation of gene expression. The correct inheritance of epigenetic patterns is crucial to cell processes while atypical DNA methylation is linked to numerous diseases, disorders and abnormalities^1,2^. DNA methyltransferase (Dnmt1) and UHRF1 (ubiquitin-like, containing PHD and RING finger domains) are recognized as the main players in the preservation of DNA methylation in mammals.

In bacteria the majority of DNA methyltransferases described are part of restriction-modification (RM) systems. A RM system consists of a restriction endonuclease and a DNA (adenine or cytosine) methyltransferase. Usually, base methylation protects host DNA from DNA cleavage by the associated endonuclease. ‘Orphan’ DNA methyltransferase genes can be found in many bacterial genomes and probably derive from ancestral RM systems that lost the cognate restriction enzyme. Additional roles in regulating several important cellular processes, such as initiation of DNA replication, DNA repair and gene regulation, were proposed for bacterial adenine methyltransferases^3–8^. The most famous examples are the adenine DNA methyltransferases Dam and CcrM. In *Escherichia coli* Dam is important for gene expression as well as other cellular processes, like DNA replication initiation and DNA repair^9–11^. In *Caulobacter crescentus* and other *Alphaproteobacteria* CcrM is essential to regulate gene expression and controls more than 10% of the genes necessary for its cell cycle progression^12^. Recently, roles in regulating gene expression were also given to orphan cytosine methyltransferases of *Helicobacter pylori* and *E. coli*. In *H*. *pylori* an orphan cytosine methyltransferase influences the expression of genes involved in motility, adhesion, and virulence^13^. In *E*. *coli*, the Dcm cytosine methyltransferase controls the expression of two ribosomal protein genes, the drug resistance transporter gene *sugE* at early stationary phase^10,14,15^ and the expression of genes associated with stationary phase^16^.

5-azacytidine (5-azaC) and 5-aza-2′-deoxycytidine (5-aza-dC) are cytosine DNA methylation inhibitors routinely used to demethylate DNA in a variety of eukaryotes to assess the consequences of cytosine DNA methylation loss^17,18^. They are nucleoside analogs that are converted intracellularly to the corresponding 5′-triphosphates upon cell entry; 5-azaC is incorporated into both RNA and DNA, whereas 5-aza-dC only into DNA^18–22^. When these analogues are incorporated, cytosine-5 DNA-dependent cytosine methyltransferases are locked on the DNA and inhibited with the consequence of decreased 5-methylcytosines in newly replicated DNA^20,21^. Recently, 5-azaC use was applied to *E*. *coli* where it was found to modulate transcriptome^23^.

Streptomycetes are Gram positive soil bacteria with CG rich genomes (70%). They are industrially very important because they produce two thirds of all clinically relevant secondary metabolites^24^. *Streptomyces coelicolor* A(3)2 strain M145 is the best-known species of the *Streptomyces* genus at both genetic and molecular level^25–27^ and it has long been considered as the model streptomycete for studying physiological (antibiotic production) and morphological differentiation. *S. coelicolor* A(3)2 M145 produces three well characterised antibiotics (actinorhodin, blue pigment, Act; undecylprodigiosin, red pigment, Red; calcium-dependent lipopeptide antibiotic, CDA), and has been described to encode for up to 30 additional secondary metabolites^28^. *S*. *coelicolor* M145 exhibits a complex developmental cycle that includes sporulation and developmentally associated programmed cell death^29,30^. In a solid culture (i.e. GYM) five different cell types are sequentially produced: the unigenomic spores, the first mycelium (MI), the second mycelium (MII), aerial cells and sporulating cells. After spore germination, a viable vegetative mycelium grows on the surface and within the agar matrix forming the first compartmentalized mycelium that undergoes a highly ordered PCD. The remaining viable segments of these hyphae enlarge and form the second multinucleated mycelium MII that comprises (i) the MII substrate that grows within the agar matrix, (ii) the aerial MII characterized by hydrophobic layers and (iii) the sporulating MII, which undergoes a second round of PCD followed by spore formation. *S*. *coelicolor* life cycle is regulated at different levels by extracellular signals and quorum sensing-related factors, multiple master regulators, and biochemical pathways, such as *bald*, *white* and *sky*^27,31,32^, but little is known regarding the effect of DNA methylation controlling differentiation.

*S*. *coelicolor* M145 has a stringent type IV restriction-modification system that cleaves exogenous methylated DNA, and for its successful transformation it is first necessary to demethylate DNA constructs in a *dam*^−^ *dcm*^−^ mutant strain of *E*. *coli*^33^. Recently, an endonuclease that binds to 5-methyl-cytosine containing DNA in all sequence contexts was characterized^34^. Years ago, the role of DNA methyltransferases in *Streptomyces antibioticus* and *S*. *coelicolor* was investigated by treating the cultures with demethylating agents and it was found that methylation could influence development and differentiation^35–37^.

In this study, we investigated whether *S*. *coelicolor* M145 genome undergoes differential DNA cytosine methylation during the growth cycle and whether treatment with a demethylating agent (5-aza-dC) could affect growth and differentiation. We found that DNA cytosine methylation is modulated during development and that demethylation impairs morphological differentiation and actinorhodin production. Thus, we searched for DNA methyltransferase genes in the genome and constructed a mutant in a putative DNA methyltransferase gene. Our data showed that in the *SCO1731::Tn5062* strain, methylation levels decreased and growth and differentiation were delayed, similarly to the effects caused by the treatment of *S*. *coelicolor* M145 with the demethylating agent. To the best of our knowledge, this is the first study that demonstrates the involvement of cytosine methylation in the control of morphological and physiological differentiation in this microorganism.

## Results

### DNA cytosine methylation varies during development of Streptomycetes

**Figure 1:**
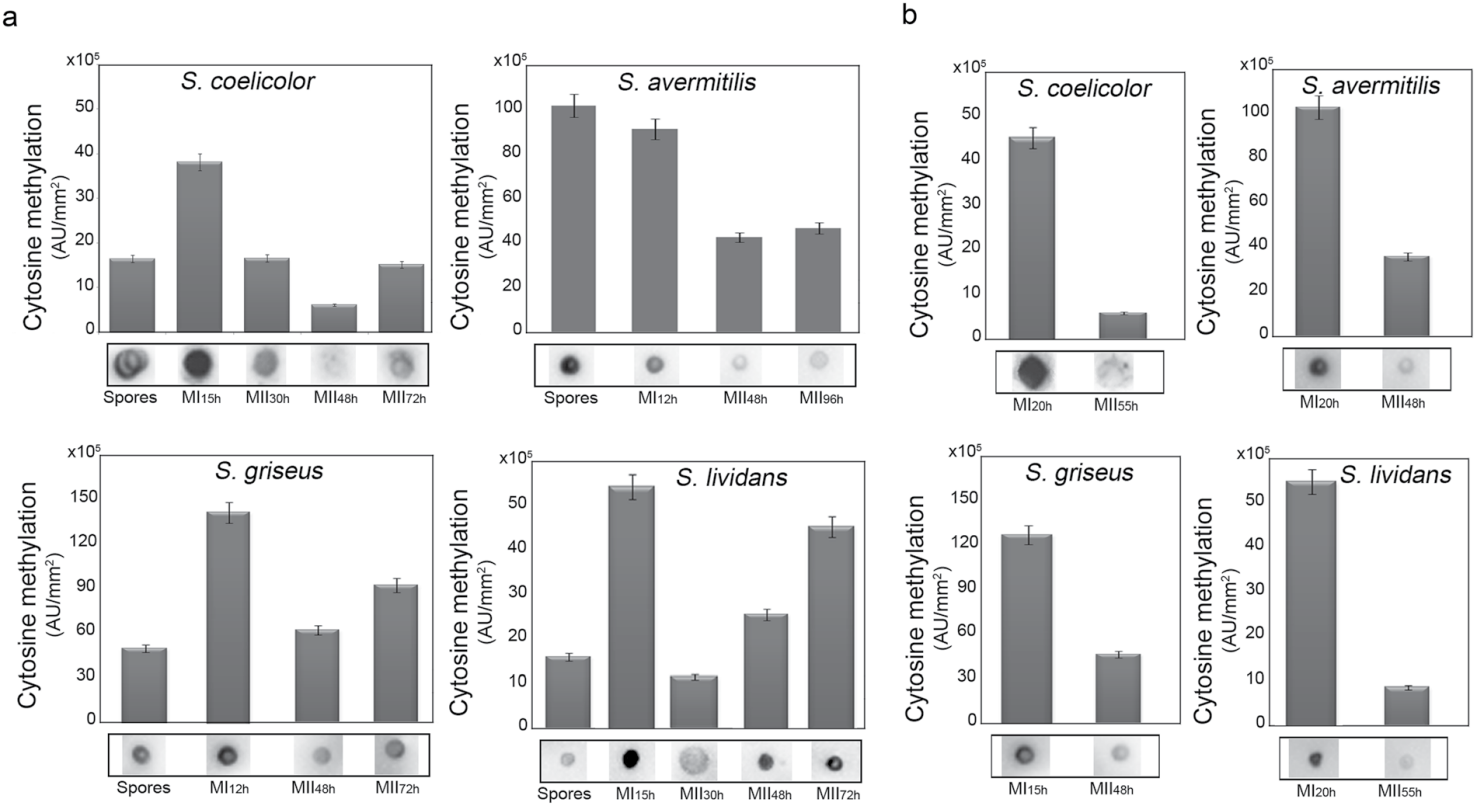
Methylation levels along the different growth phases of *S*. *coelicolor*, *S*. *lividans*, *S*. *griseus* and *S*. *avermitilis*. Genomic DNA was extracted from bacterial cultures grown both in liquid and on solid medium and analyzed by dot blot assay with antibody against 5MeC. Bars represent methylation levels in arbitrary units (AU) quantifying dot blot signal intensities. Error bars were obtained from three independent experiments. Examples of dot blots are shown below the bars. MI and MII stages are indicated. (**a**) Solid cultures. (**b**) Liquid cultures. MII_48h_ and MII_72h_ correspond to aerial and sporulating aerial hyphae, respectively. The full length blots are included as Supplementary Fig. S8.

Genomic DNA was extracted from different developmental stages of *S*. *coelicolor* M145, *S*. *avermitilis* ATCC 31267, *S*. *griseus* NBRC 102592 and *S*. *lividans 1326* and analyzed by dot blot assay using the antibody against 5-MeC (Fig. 1). To our surprise, this analysis showed that cytosine methylation is higher at the MI stage than at the MII stages in all the conditions (solid GYM cultures and sucrose-free R5A liquid medium) and species analysed (Fig. 1). Aerial hyphae (MII_48h_) showed to have the lowest levels of methylated DNA in *S*. *coelicolor* development in solid GYM cultures (Fig. 1a), while MII hyphae (MII_55h_) showed the lowest methylation levels in liquid sucrose-free R5A cultures (Fig. 1b).

*S*. *coelicolor* M145, *S*. *griseus* and *S*. *avermitilis* degrade exogenous methylated DNA, while *S*. *lividans* does not^38,39^. *S*. *lividans 1326* and *S*. *coelicolor* M145 are different in accepting methylated DNA, but they have a very similar genome and a similar development^40^. Despite of that, DNA cytosine methylation during development is comparable in all the *Streptomyces* species in all the growth phases analyzed (higher at the MI stage), indicating that (i) the cytosine methylation is conserved in the Streptomycetaceae family, (ii) has a broader role on *Streptomyces* differentiation and (iii) is not only a tag to recognize and degrade exogenous DNA.

### Effect of cytosine demethylation using 5-aza-dC on *S*. *coelicolor* M145 differentiation

Thus, to assess whether the modulation of cytosine methylation could have a role on morphological and physiological differentiation, a treatment with the hypomethylating agent 5′-aza-2′-deoxycytidine (5-aza-dC) was performed. Experiments for the set-up of the cytosine DNA demethylation treatment were carried out, as described in the methods. *Streptomyces coelicolor* M145 cultures were treated with 5 µM of the hypomethylating agent 5-aza-dC every 12 h.

**Figure 2:**
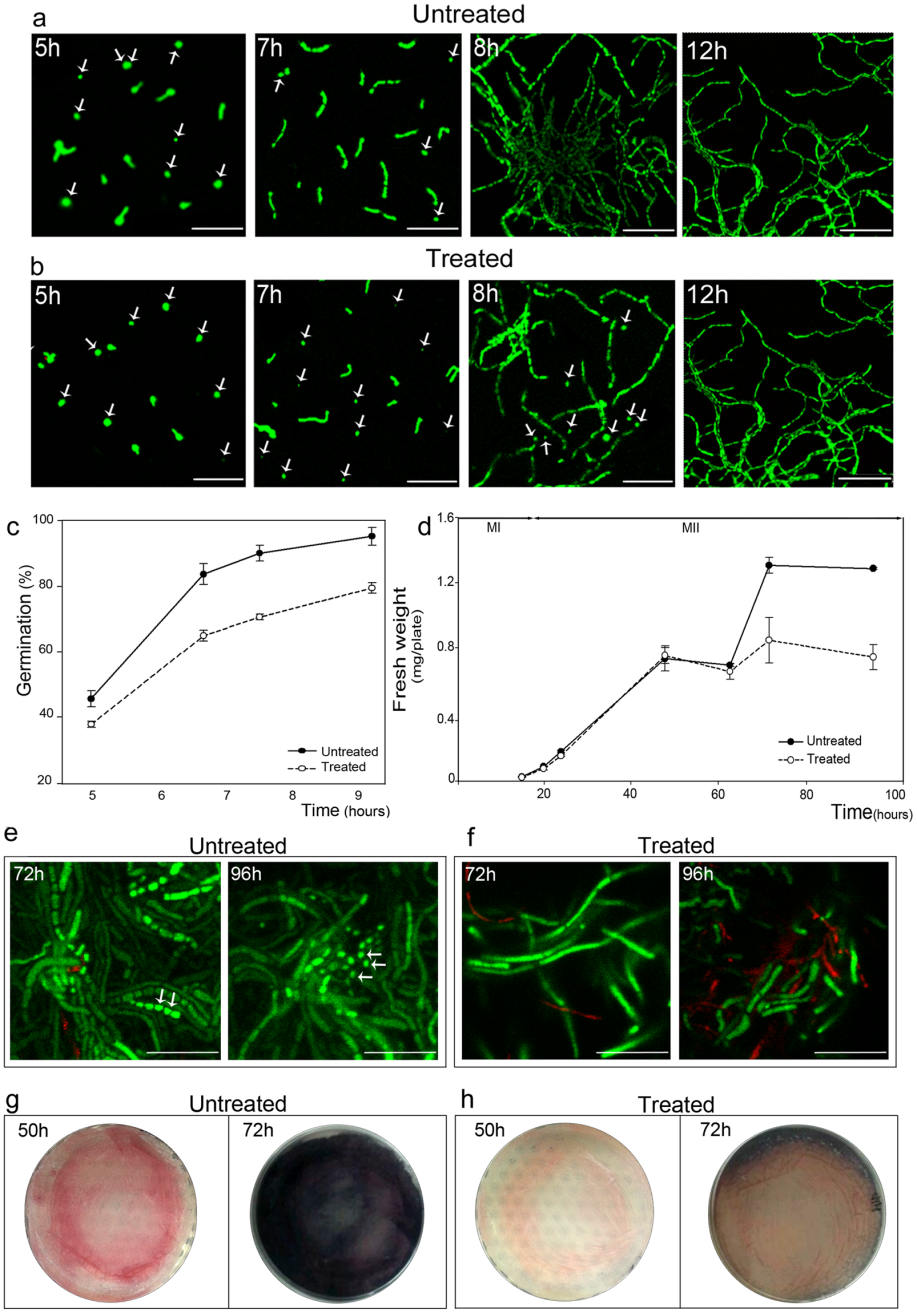
Effect of 5-aza-dC treatment on *S*. *coelicolor* morphological and physiological differentiation on solid GYM. (**a**,**b**) CLSM analysis (LIVE/DEAD Bac-Light bacterial viability kit staining) of the untreated and treated cultures after 5, 7 and 8h from seeding of the same spore stock on GYM plates with or without 5-aza-dC. (**c**) Percentage of spore germination after 5 h, 7 h, 8 h and 9 h of growth of untreated and treated cultures. (**d**) Growth curves of untreated and treated cultures. (**e**,**f**) CLSM analysis of untreated and treated cultures at 72 and 96 h. (**g**,**h**) Macroscopic view of undecylprodigiosin (red color) and actinorhodin (blue color) production of untreated and treated cultures at 50 and 72 h on GYM plates.

Analysis under confocal laser scanning microscopy (CLSM) after SYTO 9 and PI staining demonstrated that 5-aza-dC reduced spore germination up to 65% in respect to the 95% of the untreated culture after 9 h of growth. After 12 h, the spores of the treated culture were germinated as those of the untreated culture (Fig. 2a–c). Growth curves on solid medium of the untreated and the 5-aza-dC treated *S*. *coelicolor* M145 cultures revealed that there was little effect of 5-aza-dC on bacterial growth for the first 63 h; after 63 h, the treated culture grew very slowly and remained in stationary phase (Fig. 2d). At 72 h and 96 h the 5-aza-dC treated samples showed the multinucleated secondary mycelium (MII) characterized by non-septate branching non-sporulating hyphae (Fig. 2f), while the untreated culture presented spore chains and single spores (Fig. 2e). In the 5-aza-dC treated culture, the undecylprodigiosin (red pigment) and actinorhodin (blue pigment) production was decreased compared to the untreated culture (Fig. 2g,h).

**Figure 3:**
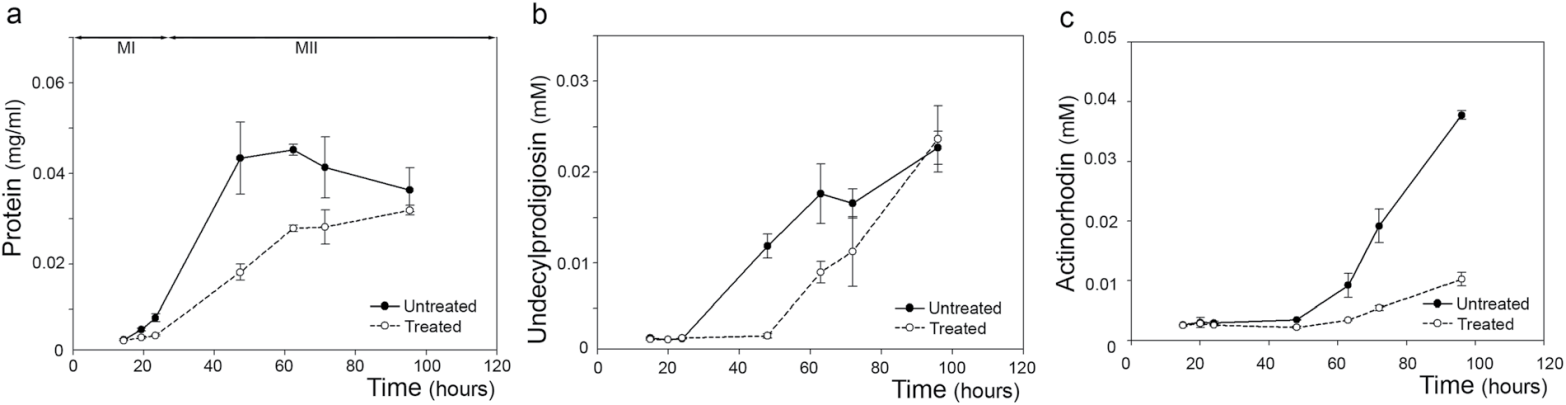
Effect of 5-aza-dC treatment in sucrose-free liquid R5A cultures. (**a**) Growth. (**b**) Undecylprodigiosin production. (**c**) Actinorhodin production. Continuous and dashed lines indicate the untreated and the treated culture, respectively.

In liquid sucrose-free R5A cultures, the addition of 5-aza-dC decreased growth rate (Fig. 3a) and delayed spore germination; indeed, ungerminated spores were still present at 20 h in the treated culture, as visualized by CLSM (Supplementary Fig. S1). In the 5-aza-dC treated liquid culture, undecylprodigiosin production started later in the treated culture, but after 100 h, the yields were similar (Fig. 3b), while actinorhodin production was 4-fold lower and started later compared to the untreated culture (Fig. 3c).

Taken together, these experiments revealed that 5-aza-dC induces a delay in morphological differentiation both in liquid and solid medium, influencing spore germination, mycelium development and sporulation; in addition, actinorhodin yield was impaired.

### Construction of a mutant in the putative DNA methyltransferase *SCO1731* gene

Bioinformatics analysis revealed that *S*. *coelicolor* M145 encodes 38 putative DNA methyltransferases (Table 1). The expression profile of these putative DNA methyltransferases was compared to the transcriptomic data, previously obtained using the same growth media^29^. This search revealed that *SCO1731* displayed the highest transcription level among the putative methyltransferase genes in MI and has orthologs in *S*. *lividans* 1326 genome (100% identity), *S*. *avermitilis* ATCC 31267 (79%) and *S*. *griseus* strain NBRC 102592 (64%).

**Table 1:** List of putative methyltransferases annotated in *S. coelicolor* genome (StrepDB - The Streptomyces Annotation Server).

Putative methyltransferase	Log 2 Ratio MII_24h_/MI	Expression phase
*SCO1731*	−1.8623	MI
*SCO0190*	−1.3723	MI
*SCO4504*	−1.298	MI
*SCO1969*	−1.1342	MI
*SCO7445*	−0.8099	MI
*SCO5972*	−0.6938	MI
*SCO0408*	−0.5595	MI
*SCO2098*	−0.3133	MI
*SCO5895*	−0.3038	MI
*SCO3545*	−0.2817	MI
*SCO2317*	−0.2732	MI
*SCO2814*	−0.2385	MI
*SCO3215*	−0.2133	MI
*SCO7055*	−0.1974	MI
*SCO2170*	−0.1867	MI
*SCO2670*	−0.1226	MI
*SCO5589*	−0.1108	MI
*SCO1555*	−0.0979	MI
*SCO5094*	−0.0884	MI
*SCO6844*	−0.0768	MI
*SCO0594*	−0.0681	MI
*SCO0760*	−0.0136	MI
*SCO0648*	0.0093	MII
*SCO3744*	0.1059	MII
*SCO2338*	0.1692	MII
*SCO6541*	0.2415	MII
*SCO1162*	0.3867	MII
*SCO5146*	0.4161	MII
*SCO0929*	0.5308	MII
*SCO6549*	0.6532	MII
*SCO6928*	0.7393	MII
*SCO3452*	0.7782	MII
*SCO0835*	0.7845	MII
*SCO0826*	0.8163	MII
*SCO7452*	0.8841	MII
*SCO5257*	1.3063	MII
*SCO0392*	2.0070	MII
*SCO0995*	2.4922	MII

Ratios of log_2_ of gene expression between MII24 h/MI are reported. A negative ratio indicates that the gene is more transcribed in MI, a positive one that is more transcribed in MII 24 h. The ratios were taken from Yagüe *et al*.^29^.

**Figure 4:**
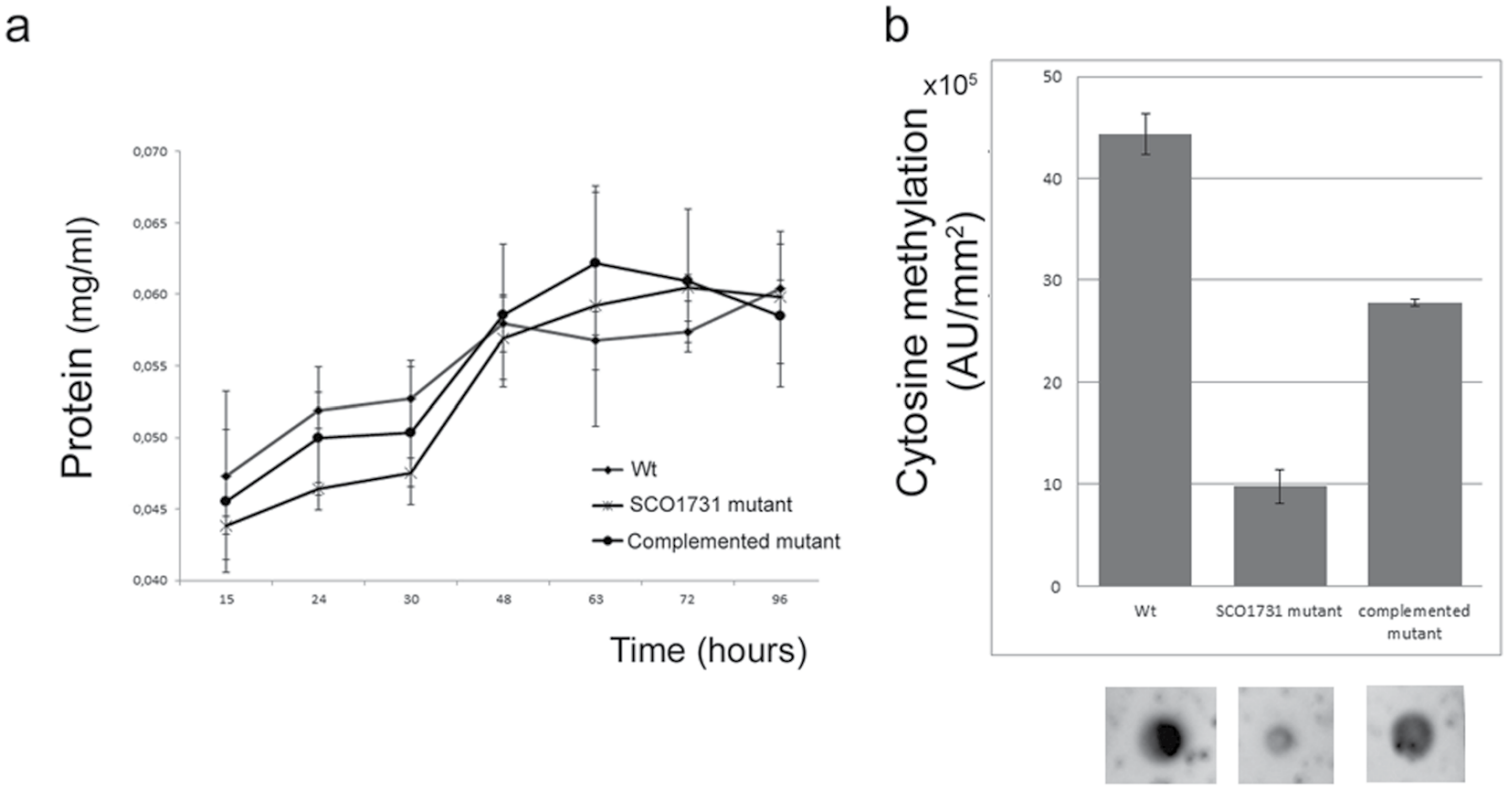
Growth and cytosine methylation of the *S*. *coelicolor* wild-type strain, the *SCO1731::Tn5062* mutant and the complemented *SCO1731::Tn5062* mutant, in sucrose-free R5A cultures. (**a**) Growth curves. (**b**) cytosine methylation levels at 20-hours (MI). AU indicate arbitrary units of methylation levels. The full length blots are included as Supplementary Fig. S9.

Thus, since methylation levels were found higher in MI we evaluated if this gene is important for cytosine methylation by generating a mutant using a cosmid containing the gene interrupted by the transposon Tn5062^41^. In liquid sucrose-free R5A cultures, disruption of *SCO1731* did not significantly alter the growth kinetics (Fig. 4a) and spore germination (Supplementary Fig. S1) of the *SCO1731::Tn5062* mutant strain, indicating that this gene is not critical for bacterial growth under the used conditions.

Dot blot analysis demonstrated that the cytosine methylation levels were strongly reduced in the *SCO1731::Tn5062* mutant strain at 20 h (MI) (from 44, 38 × 10^5^ ± 2 × 10^5^ to 9, 8 × 10^5^ ± 1, 6 × 10^5^) (Fig. 4b).

**Figure 5:**
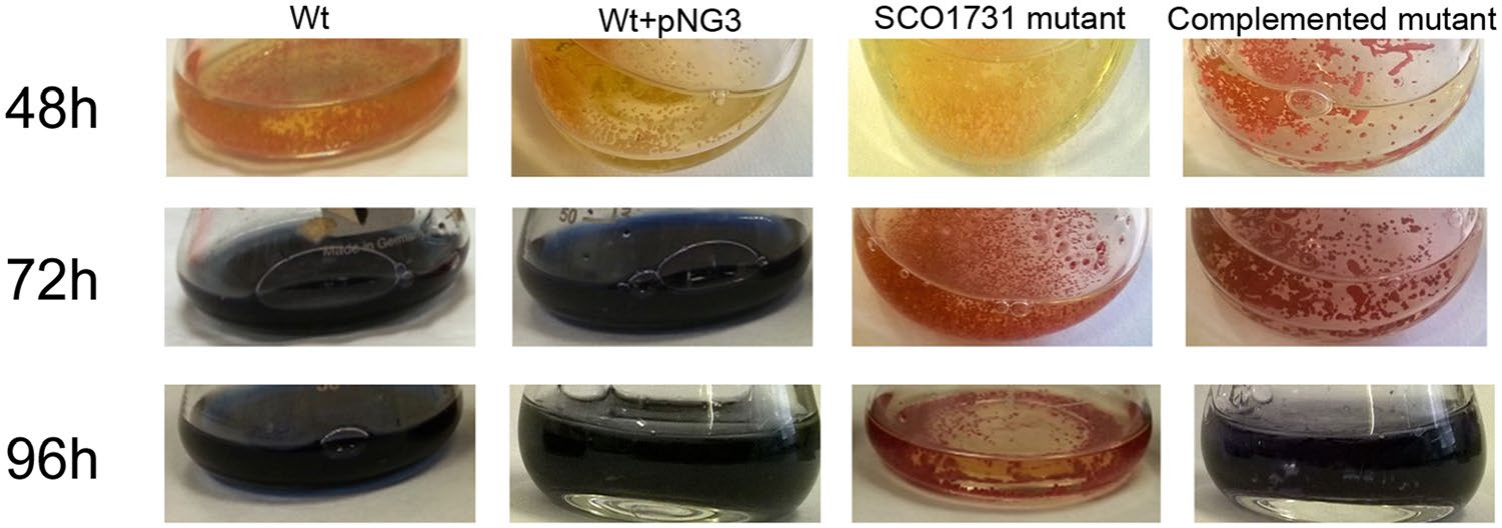
Actinorhodin production in sucrose-free R5A cultures of the *S*. *coelicolor* wild-type strain, the *SCO1731::Tn5062* mutant and the *SCO1731::Tn5062* complemented strain. Macroscopic view of laboratory flasks is shown at different developmental time points (48 h, 72 h and 96 h). Blue color corresponds to actinorhodin; red colour corresponds to undecylprodigiosin.

Conversely, the *SCO1731::Tn5062* mutant had a slight effect on cytosine methylation after 55 h of growth (MII) (Supplementary Fig. S2). Actinorhodin (blue pigment) was not observed in the *SCO1731* mutant strain in liquid sucrose-free R5A even after 96 h of growth (Fig. 5), revealing that the mutant is impaired in actinorhodin production. *SCO1731::Tn5062* mutant cultures produced undecylprodigiosin (red color) (Fig. 5).

**Figure 6:**
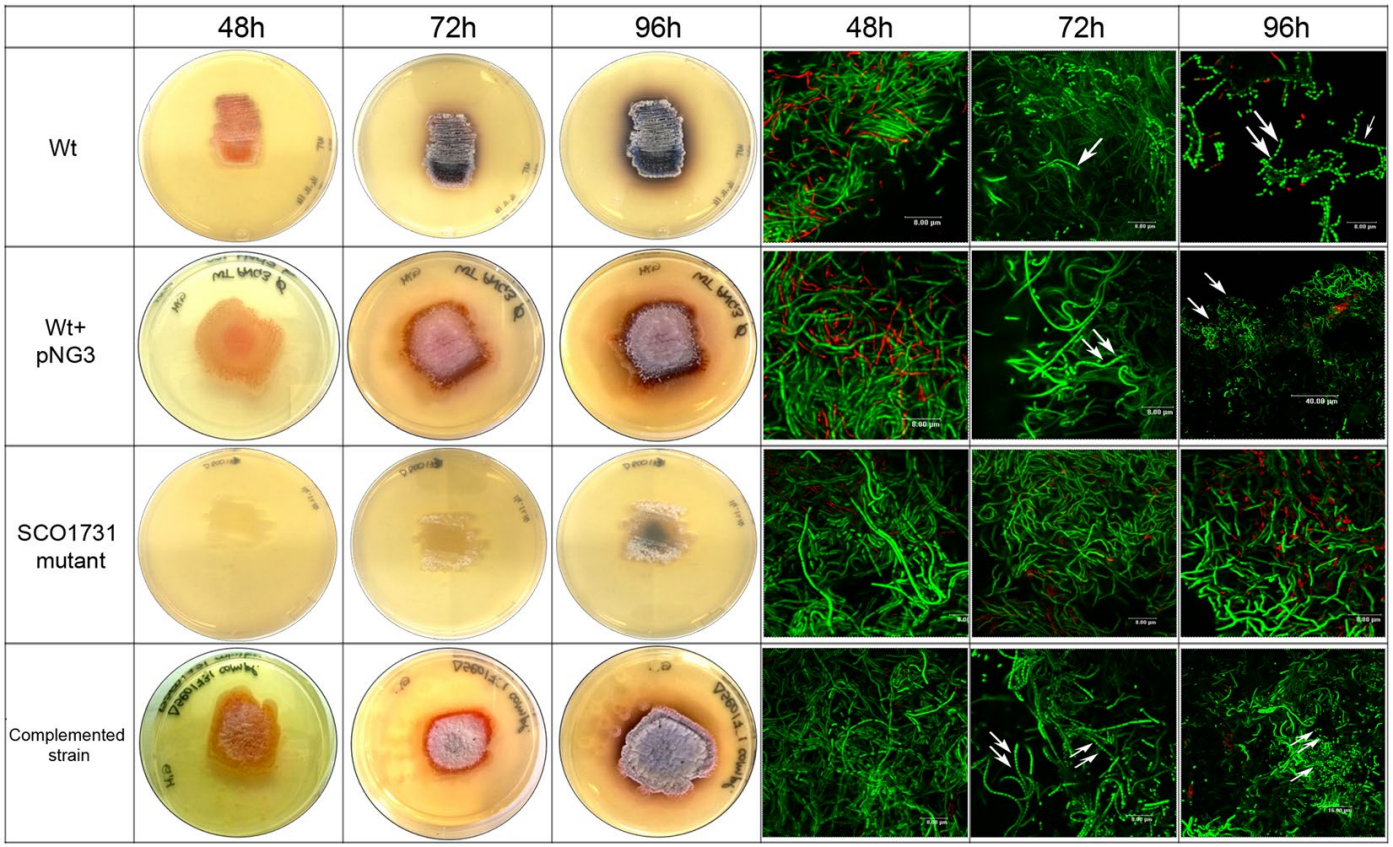
Morphological differentiation of the *S*. *coelicolor* wild-type strain, the *SCO1731::Tn5062* mutant and the *SCO1731::Tn5062* complemented strain on GYM plates. Macroscopic view (left panels); CLSM images (right panels) taken after staining the cells with the LIVE/DEAD Bac-Light bacterial viability kit. Arrows indicate spore chains.

Likewise, the inactivation of *SCO1731* caused a marked delay in morphological and physiological differentiation on solid GYM (Fig. 6): aerial mycelium formation started at 96 h in the *SCO1731::Tn5062* mutant strain compared to the 48 h in the wild-type strain; spore chains were not formed up to 96 h in the mutant strain compared to 72 h in the wild-type strain; actinorhodin (blue color) and undecylprodigiosin (red color) were strongly reduced in the mutant strain.

These results indicate that *SCO*1731 is responsible for methylation of cytosine in MI and it is involved in the regulation of actinorhodin production and morphological differentiation.

A complemented strain harboring a copy of the *SCO1731* ORF and its upstream region large enough to include the promoter region, was generated. In the *SCO1731*_compl strain, the methylation levels were restored to 70% of the wild-type methylation level (Fig. 4b), the morphological development (aerial mycelium and sporulation) was fully restored (Fig. 6), and actinorhodin production was reestablished at 96 h, but not at 72 h (Fig. 5). The control strain, containing a copy of the empty integrative vector used for complementation (*S*. *coelicolor* + pNG3), showed a normal antibiotic production profile (Fig. 5) and development (Fig. 6), excluding an effect of pNG3 integration on the phenotypes observed in the *SCO1731*_compl strain.

## Discussion

Cytosine and adenine methylation are epigenetic mechanisms to control gene expression in eukaryotic and prokaryotic organisms, respectively. While adenine methylation has been largely studied in many bacterial systems and it was shown to influence crucial vital processes, such as bacterial cell cycle, only a few studies have so far been published on cytosine methylation in bacteria, mostly in *Escherichia coli*^4,5,16,23^. Years ago, some attempts to find a role for DNA methyltransferases in *S*. *antibioticus* and *S*. *coelicolor* were reported. These studies applied different compounds known to block cytosine methylation (e.g. 5-azacytidine and sinefungin), but no clear role for cytosine methylation was established^35–37^. Here, we demonstrate that methylation levels are modulated throughout the growth cycle in both, solid and liquid media. This result is of particular interest, since *S*. *coelicolor* transformation efficiency depends on the methylation status of exogenous DNA^38–40^. We also demonstrate that the hypo-methylating agent 5′-aza-2′-deoxycytidine (5-aza-dC) causes a delay in spore germination, aerial mycelium differentiation and sporulation in solid medium and affects growth and spore germination in liquid medium; in addition, actinorhodin production is massively impaired in both solid and liquid media; differently, undecylprodigiosin production is retarded, but the yields in the treated cultures are similar to the untreated ones. Unfortunately, our results cannot be compared to previous reports using hypo-methylating agents in *Streptomyces*^35–37^, since 5-azacytidine is incorporated into both DNA and RNA, and sinefungin is an inhibitor of SAM-dependent cytosine and adenine methyltransferase, while in our experiments we used 5-aza-2′-deoxycytidine that is only incorporated in DNA. Thus, the effect we noticed is essentially due to cytosine methylation in the genome.

We demonstrate that *SCO1731* codes for a cytosine methyltransferase involved in the cytosine methylation accompanying *Streptomyces* differentiation. In the *SCO1731::Tn5062* mutant strain, cytosine methylation was reduced to 22% compared to the parental strain during the MI stage. Our results cannot rule out that other methyltransferases may be responsible for residual methylation nor that other methyltransferases may be expressed following *SCO1731* activation in a cascade manner. In fact, 38 genes coding for putative methyltransferases are present in *S*. *coelicolor* genome, making this a reasonable hypothesis. Among them, *SCO1731* was transcribed in MI at higher levels, when cytosine methylation is higher than in other growth stages both in liquid and solid medium. Moreover, the *SCO1731::Tn5062* mutant shows the same phenotype observed in the 5-aza-2′-deoxycytidine treated cultures regarding the effect on actinorhodin production, aerial mycelium differentiation and sporulation, that resulted impaired in the *SCO1731::Tn5062* mutant and the 5-aza-2′-deoxycytidine treated cultures; differently, the delay observed in undecylprodigiosin production, spore germination and growth in the 5-aza-2′-deoxycytidine treated cultures was not observed in the *SCO1731::Tn5062* mutant. This further indicates that *SCO1731* is not the only methyltransferase participating in the regulation of *Streptomyces* development. Interestingly, the complemented mutant strain restored partially the methylation levels to 70% and if on the one hand this was sufficient to restore the correct morphological development, on the other one said methylation level was not sufficient to re-establish the onset of the actinorhodin production, further supporting that some other cellular events do occur. This kind of multilevel regulation would be not far from other bacterial systems, better investigated for influence of DNA methylation on gene expression. For instance, in *C*. *crescentus* many genes are controlled by the CcrM methyltransferase and are also co-regulated by other global cell cycle regulators, demonstrating extensive cross talk between DNA adenine methylation and the complex regulatory network governing cell cycle progression^12^.

Given that *S*. *coelicolor* undergoes a complex life cycle with two programmed cell death events, we hypothesized that the hypermethylation of genomic DNA in MI could be a signal that activates DNA cleavage in some cells leading to cell death and allowing a controlled life cycle. In *E*. *coli*, it was demonstrated that cell death occurs upon an induced cytosine hypermethylation of the genome^42–44^. Even if this were the case, it would still be difficult to explain how adjacent cells perceive different stimuli and follow different fates^29^. If this hypothesis were correct, PCD and differentiation of *S*. *coelicolor* would have to be blocked after 5-aza-dC treatment and in the *SCO1731::Tn5062* mutant strain, and this is not the case. Notwithstanding, it is possible that 5-aza-dC effect is temporary and that other methyltransferases or pathways are activated in the *SCO1731::Tn5062* mutant strain. An alternative hypothesis about the biological role of cytosine methylation, is that it influences gene expression, as it occurs in other systems^14–16,23,45^. Methylation of cytosines in eukaryotic promoters leads to repression of transcription and to an activation when it affects gene bodies^45^. In *E*. *coli* the absence of the *dcm* gene leads to a differential expression of 510 genes, i.e. two ribosomal protein genes and the drug resistance transporter gene *sugE*, at stationary phase^14–16^. Future work will be addressed to identifying SCO1731 target genes and to comparing the methylome of *S*. *coelicolor* parental and the *SCO1731::Tn5062* mutant strains.

Overall, this is the first report that correlates DNA cytosine methylation with differentiation in *S*. *coelicolor* and attributes a DNA methyltransferase function to the *SCO1731* gene. Our results show that both, the treatment with 5-aza-2′-deoxycytidine as well as the inactivation of the *SCO1731* gene, result in a strong impairment in morphological differentiation (delay in aerial mycelium and sporulation) and an impediment in actinorhodin production. Our results reveal, for the first time, that epigenetics, through methylation of cytosines, control morpho-physiological differentiation in *S*. *coelicolor* unveiling new levels of complexity of gene expression and regulation in this microorganism.

## Methods

### Bacterial strains and media

Bacterial strains, plasmids and cosmids are listed in Table 2 The strains were grown in sucrose-free R5A, GYM and SFM, and maintained by following procedures, reported in Manteca *et al*.^46^. *Escherichia coli* strains were grown at 37 °C in solid or liquid 2xYT^47^ medium supplemented with the appropriate antibiotics.

**Table 2:** Bacterial strains, plasmids and cosmids used in this study.

	Description	Origin or reference
**Bacterial strains**		
*S*. *coelicolor* M145	SCP1^−^SCP2^−^	^39^
*S*.*coelicolor SCO1731::Tn5062*	*SCO1731::Tn5062*, Am^R^	This study
*Streptomyces lividans* 1326	SCP1^-^SCP2^-^	^39^
*Streptomyces griseus* NBRC 102592		NBRC
*Streptomyces avermitilis* ATCC 31267		ATCC
*Escherichia coli DH10B*	F- *mcr*A Δ(*mrr*-*hsd*RMS-*mcr*BC) φ80*lac*ZΔM15 Δ*lac*X74 *rec*A1 *end*A1 *ara*D139 Δ(*ara*, *leu*)7697 *ga*/U *ga*/K λ- *rps*L *nup*G	Invitrogen
*Escherichia coli* ET12567/pUZ8002	F-*dam*-13::Tn9 *dcm*6 *hsd*M *hsd*R *rec*F143 zjj201::Tn10 *gal*K2 *gal*T22 *ara*14 *lac*Y1 *xyl*-5 *leu*B6	^39^
**Plasmid/Cosmid**		
pCR™-Blunt II-TOPO®	Zero Blunt® TOPO® PCR Cloning Kit, Km^R^	Invitrogen
pNG3	*bla* cloned into pNG1/*HindIII*/*AvrII* Hyg^R^, Amp^R^	^51^
pQM5062	Plasmid containing *eGFP* Tn5062	^52^
pNG3-1731compl	pNG3 plasmid containing the ORF of *SCO1731*	This study
I11.2.G06	I11 cosmid carrying I11.2.G06 transposant	^41^

### Dot Blot assay

Genomic DNAs were extracted by salting out procedures, as described in Lo Grasso *et al*.^48^. Dot blot assay was performed following the protocol described in Caracappa *et al*.^49^. Briefly, genomic DNA was denatured at 95 °C for 10 min, spotted on nitrocellulose filter (Hybond ECL, GE Healthcare Life Sciences) and fixed by UV (2 cycles at 700 J). The spotted DNA was detected by staining the filter with 0.02% (w/v) methylene blue in 0.3 mol/L sodium acetate (pH 5.2). After removal of the staining solution, the methylated cytosines were detected using the anti-5-methylcytosine mouse antibody (Calbiochem) and the secondary goat anti-mouse IgG-H&L chain specific peroxidase conjugate (Calbiochem). The images were taken using Chemidoc (Chemi Hi sensitivity) and SuperSignal®West Femto maximum sensitivity substrate (Life Technologies). Spots of the same area were manually labelled and quantified by Molecular Imager ChemiDoc XRS System Biorad. Percentage of methylation level was reported as arbitrary units per mm^2^ (AU/mm^2^). The experiments were performed at least twice and in triplicate.

Genomic DNAs, extracted from *Escherichia coli DH10B* and *Escherichia coli ET12567/pUZ8002* strains, were used as positive and negative control, respectively. The experiments were performed at least twice and in triplicate.

### 5-aza-dC treatment

Preliminary experiments were performed to set up the demethylation. The amount of 5-aza-dC (Sigma) to add to the medium was chosen after checking the effect of increasing concentrations of the drug on the cells on solid medium GYM (Supplementary Fig. S3). 5-azadC is reported to have a half-life of 20h-24h under conditions of physiological temperature and neutral pH^50^, so the treatment was repeated every 24 h, from 0 to 96 h. A control experiment was done in parallel using DMSO (the solvent of 5-aza-dC). 5 µM 5-aza-dC was the highest concentration in which the cells were still viable, while 10 and 15 were lethal for the cells, indeed a halo of growth inhibition was present. This is in accordance with results reported for *E*. *coli*^23^.

In addition, a treatment was carried out to liquid cultures every 12 and 24 h, from 0 to 36 h. A control experiment was done in parallel using DMSO. The efficiency of demethylation was evaluated after 48 h of growth of *S*. *coelicolor* in the presence of 5 µM of 5-azadC added every 24 h and 12 h, by dot blot analysis (Supplementary Fig. S4). The results revealed that the efficiency of the treatment carried out every 24 h was 72%, while every 12 h was 99, 5%. Thus, for the demethylation experiments treatment was carried out with 5 µM 5-azadC added every 12 h.

### Confocal laser scanning microscopy analysis (CLSM)

Culture samples were processed for microscopy at different incubation time points following the protocol reported in Manteca *et al*.^46^. Cells were stained with the LIVE/DEAD Bac-Light bacterial viability kit (Invitrogen), that contains the SYTO 9 green fluorescent stain for labelling all the cells and the non-cell-permeating nucleic acid stain (propidium iodide, PI) for detecting the dead cells. The samples were observed under a Leica TCS-SP2-AOBS confocal laser-scanning microscope at a wavelength of 488 nm and 568 nm excitation and 530 nm (green) or 640 nm (red) emissions. More than 30 images were analyzed for each developmental time point in a minimum of three independent cultures.

### Antibiotic quantification

To measure actinorhodin (intracellular and extracellular), cells were broken in their culture medium by adding KOH 0.1 N. Cellular debris was discarded by centrifugation, and actinorhodin was quantified spectrophotometrically with UV/visible spectrophotometer, applying the linear Beer-Lambert relationship to estimate concentration (Ɛ640 = 25,320). Undecylprodigiosin was measured after vacuum drying of the mycelium, followed by extraction with methanol, acidification with HCl (to 0.5 N), and spectrophotometric assay at 530 nm, again using the Beer-Lambert relationship to estimate concentration (Ɛ530 = 100,500). Reproducibility has been corroborated by at least three independent cultures at various developmental time points.

### Disruption of the *SCO1731*

To generate the *SCO1731::Tn5062* mutant, the cosmid I11.2.G06 containing a copy of the gene interrupted by the transposon Tn5062^41^ was used. It contains the apramycin and kanamycin resistance cassettes in the transposon and in the cosmid, respectively. After transformation by interspecific conjugation with *E*. *coli* ET12567/pUZ8002 as a donor strain, 8 apramycin resistant colonies were obtained. Genomic DNA was extracted from 4 mutants and analyzed by PCR for the presence of apramycin (~1.3 kb) and the absence of kanamycin (0.9 kb) resistance cassette. The following primers were used Kana_F 5′-GATGGCTTTCTTGCCGCC-3′ and Kana_R 5′-TCGGTCATTTCGAACCCC-3′, Apra_F 5′-CGGGGTACCCTCACGGTAACTGATGCC-3′ and Apra_R 5′-ATTTTAATGCGGATGTTGCG-3′ to amplify apramycin or kanamycin resistance cassette, respectively. Two samples (*SCO1731*-3 and *-*4) had the expected profile (Supplementary Fig. S5) and they were analyzed by Southern Blot using genomic DNA digested with *SalI* and Tn5062 as a probe.

The expected restriction profile of the mutant is shown in the Supplementary Fig. S6. Southern blot analysis revealed that both putative mutants *SCO1731::Tn5062* had the expected restriction profile, two bands of approximately 2.8 and 2.7 kb.

### Complementation of *SCO1731::Tn5062* mutation

A copy of *SCO1731*, placed under the control of its promoter was amplified via PCR using Phusion High-Fidelity DNA Polymerase (Thermo), using the primers 1731_SpeI F 5′-GGACTAGTTGGCTGCCTCCTTACGGAT-3′ and 1731_compl R 5′-AAGATATCGTCTGGACGAGGACGAGTTC-3′ and was then cloned into pCR™-Blunt II-TOPO®. The sequences were checked via DNA sequencing using the M13 universal primers prior to subcloning them into pNG3^48^ constructing the plasmid pNG3-1731compl (Table 2). The plasmid was used to transform the mutant *SCO1731* strain by interspecific conjugation generating the *SCO1731*_compl strain. Thus, 32 colonies of putative *SCO1731*_compl strain were obtained after the growth in SFM with hygromycin. After 3 passages of these colonies on GYM with hygromycin, genomic DNA was extracted by 10 putative complemented strains and analyzed by PCR using the primers *SCO*4848_F 5′-CGTCGATCCCCTCGGTTG-3′ and *SCO*4848_R. 5′-GAGCCGGGAAAGCTCATTCA-3′. These primers amplified a fragment of 617 bp only if pNG3 is integrated at the *attB* site of *SCO4848*. Six of eight samples had the expected profile (Supplementary Fig. S7) and only the *SCO1731*_compl-8 was used for further experiment.

## Electronic supplementary material

Supplementary figures
