# Supplementary material for: The SCO1731 methyltransferase modulates actinorhodin production and morphological differentiation of Streptomyces coelicolor A3(2)
Source: Sci Rep. 2018 Sep 12;8:13686. doi: 10.1038/s41598-018-32027-8 (PMC6135851; doi:10.1038/s41598-018-32027-8)

The *SCO*1731 methyltransferase modulates actinorhodin production and morphological differentiation of *Streptomyces coelicolor* A3(2)

Annalisa Pisciotta[1], Angel Manteca[2], Rosa Alduina[1*]

Affiliations

[1] Department of Biological, Chemical and Pharmaceutical Sciences and Technologies (STEBICEF), Università degli Studi di Palermo, Viale delle Scienze Bd.16, 90128 Palermo, Italy. annalisa.pisciotta86@gmail.com

[2] Área de Microbiología, Departamento de Biología Funcional and IUOPA, Facultad de Medicina, Universidad de Oviedo, 33006 Oviedo, Spain. mantecaangel@uniovi.es

[*]**Corresponding author**: Rosa Alduina, Department of Biological, Chemical and Pharmaceutical Sciences and Technologies (STEBICEF), Università degli Studi di Palermo, Viale delle Scienze Bd.16, 90128 Palermo, Italy. valeria.alduina@unipa.it. +3909123897306; Work fax number: 091 6577210

Supplementary Fig. S1. CLSM of bacterial suspension of the wild-type strain, 5-aza-dC treated culture and the mutant strain after 20h of growth in liquid medium R5A. White arrows indicate ungerminated spores.

Supplementary Fig. S2. Percentage of cytosine methylation level of the wild-type strain and the *SCO1731::Tn5062* mutant strain after 20h and 55 of growth in liquid medium R5A. Methylation level of wild-type strain after 20h of growth was arbitrarily considered as 100%.

Supplementary Fig. S3. Effect of increasing concentrations of 5-aza-dC (0-15 μM) on *S. coelicolor* solid cultures, 0= control with DMSO. The pictures were taken after 50h of growth.

Supplementary Fig. S4. Percentage of demethylation efficiency after 5-aza-dC treatment carried out every 12h and 24h.

Supplementary Fig. S5. PCR products derived from A (apramycin) and K (Kanamycin) primer sets were resolved using a 0.8% agarose gel in 1X TBE buffer. Lane numbers correspond to genomic

DNA from 4 putative *SCO1731::Tn5062* clones. 5A: apramycin positive control; 5K: kanamycin positive control.

Supplementary Fig. S6. Southern blot analysis of 2 putative mutants. 1: *SalI*-digested cosmid; 2-3: *SalI*-digested genomic DNA of two *SCO1731::Tn5062* clones positive to PCR**.** M: DNA molecular weight Marker II (Roche). pQM5062 was used as a probe.

Supplementary Fig. S7. PCR products, derived from putative *SCO1731*_compl strains (lanes 1-8) were separated using a 0.8% agarose gel in 1X TBE buffer. 9: negative control; M DNA molecular weight Marker III (Roche).

Supplementary Fig. S8. Dot blots showing the cytosine methylation level of *S. coelicolor*, *S. avermitilis, S. griseus* and *S. lividans* genomic DNA, extracted after the time points indicated. MII_{48h} and MII_{72h} correspond to aerial and sporulating aerial hyphae, respectively.

Supplementary Fig. S9. Dot blot showing the cytosine methylation level of wild-type strain (A), the *SCO1731::Tn5062* mutant strain (B) and the complemented strain (C) genome after 20h of growth. (D) represents the negative control.

Fig. S1

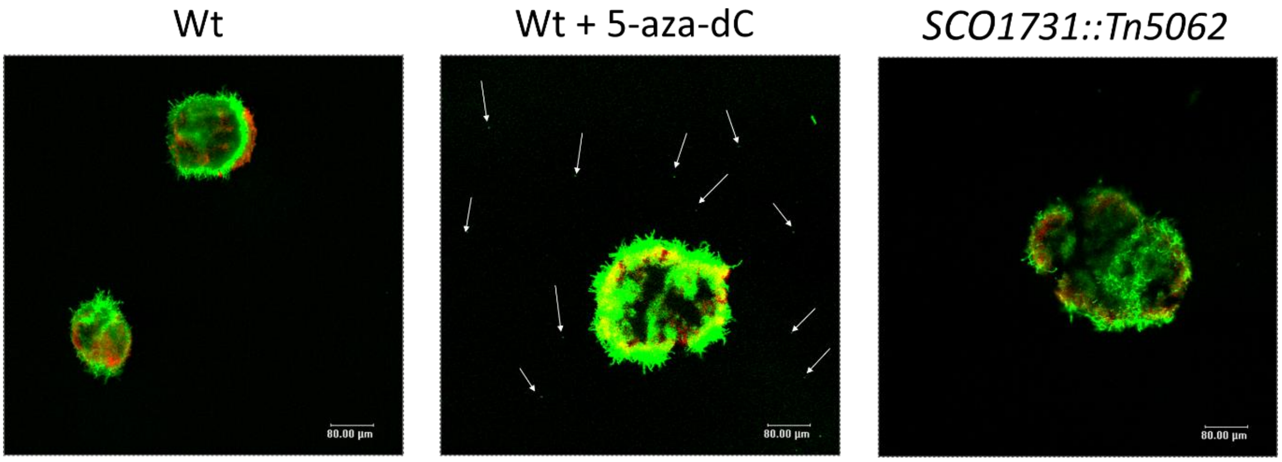

Fig. S2

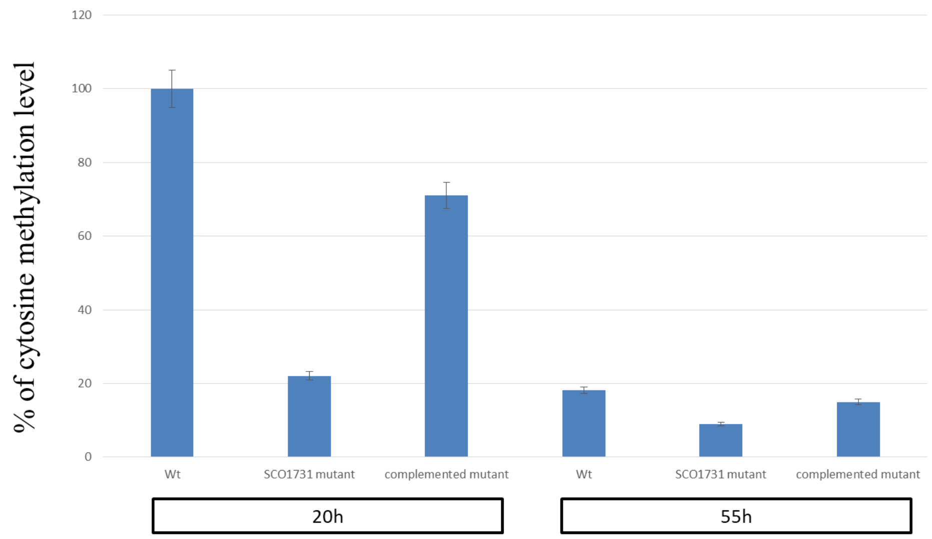

% of cytosine methylation level
120
100
80
60
40
20
0
Wt
SCO1731 mutant
complemented mutant
Wt
SCO1731 mutant
complemented mutant
20h
55h

Fig. S3

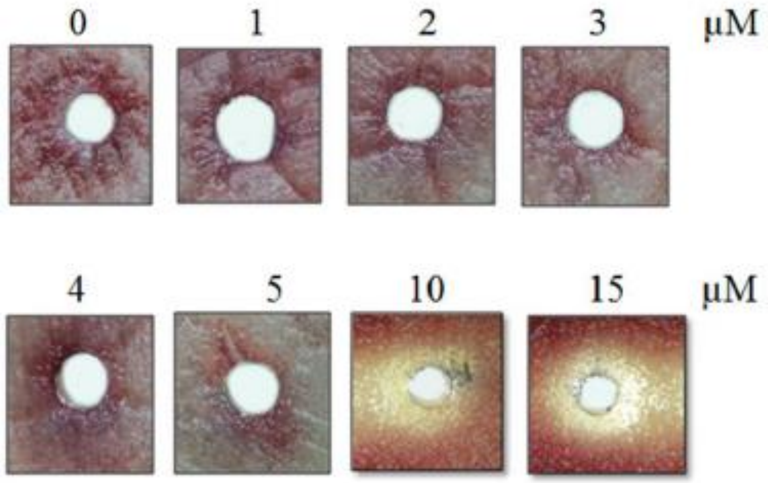

0
1
2
3
μM
4
5
10
15
μM

Fig. S4

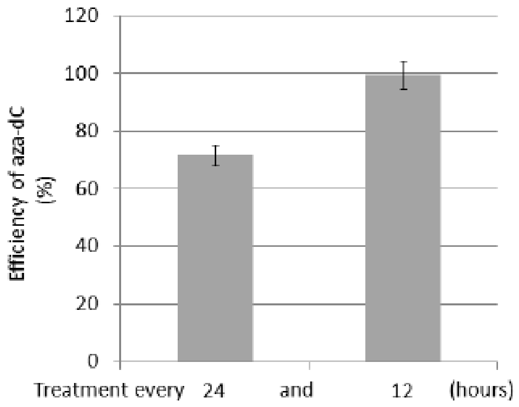

Fig. S5

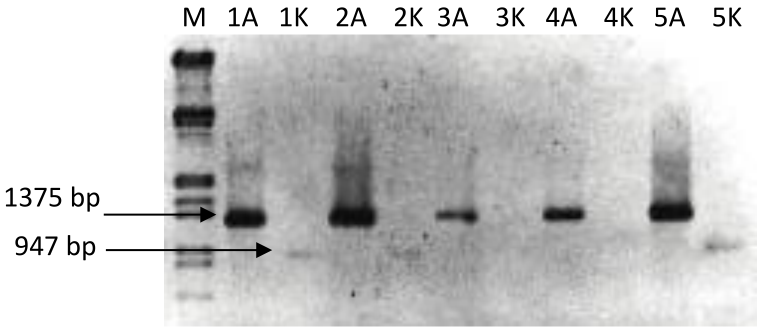

Fig. S6

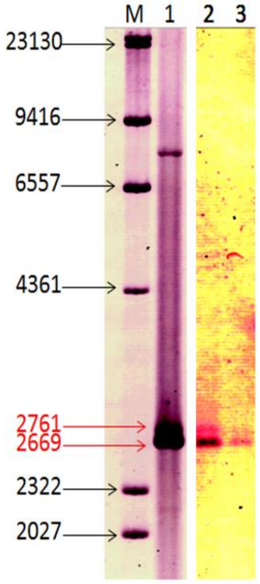

Fig. S7

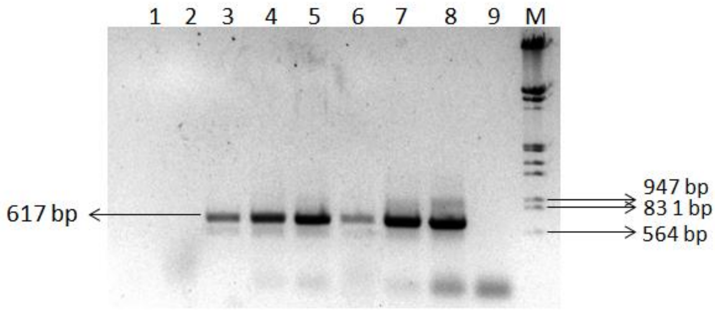
1 2 3 4 5 6 7 8 9 M
617 bp
947 bp
83 1 bp
564 bp

Fig. S8

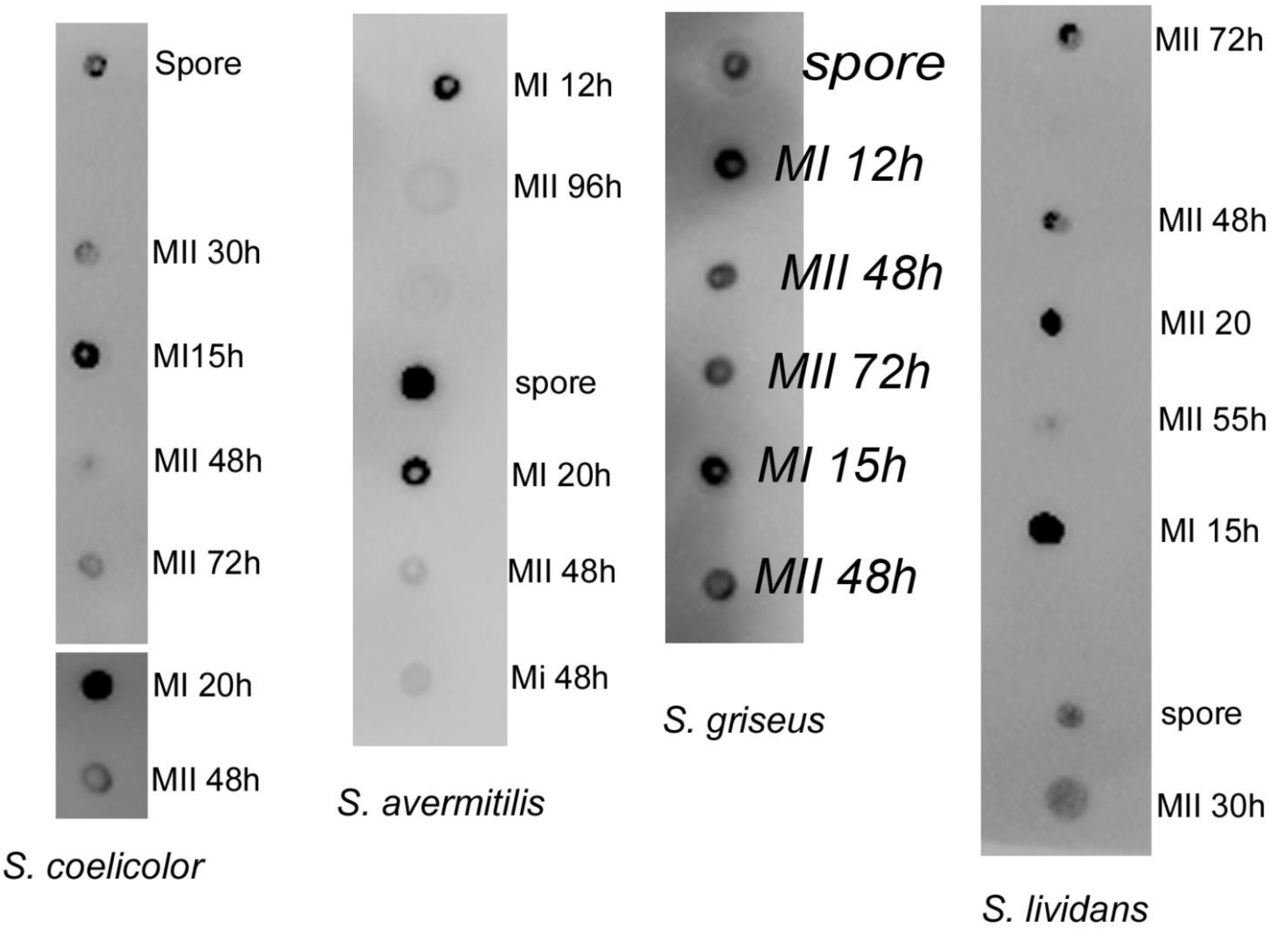
Spore
MII 30h
MI15h
MII 48h
MII 72h
MI 20h
MII 48h
S. coelicolor
MI 12h
MII 96h
spore
MI 20h
MII 48h
Mi 48h
S. avermitilis
spore
MI 12h
MII 48h
MII 72h
MI 15h
MII 48h
S. griseus
MII 72h
MII 48h
MII 20
MII 55h
MI 15h
spore
MII 30h
S. lividans

Fig. S9

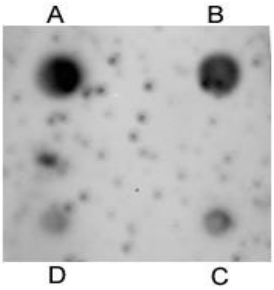

Supplement: Supplementary file 1 — Supplementary figures [file 41598_2018_32027_MOESM1_ESM.pdf]
